# Lamin B receptor plays a key role in cellular senescence induced by inhibition of the proteasome

**DOI:** 10.1002/2211-5463.12775

**Published:** 2020-01-06

**Authors:** Atsuki En, Yuki Takauji, Kensuke Miki, Dai Ayusawa, Michihiko Fujii

**Affiliations:** ^1^ Graduate School of Nanobioscience Yokohama City University Japan; ^2^ Ichiban Life Corporation Yokohama Japan

**Keywords:** autophagy, LBR, proteasome, protein accumulation, senescence, unbalanced growth

## Abstract

Cellular senescence is a terminal growth arrest phenomenon in mammalian cells. Coordinated regulation of protein synthesis and degradation is required to maintain protein homeostasis in cells; however, senescent cells exhibit decreased activity of the proteasome, a major cellular proteolytic machinery, with an accumulation of proteins. Indeed, we showed that MG132, a proteasome inhibitor, induced cellular senescence through an accumulation of proteins in human cells. We then investigated the mechanisms of cellular senescence induced by protein accumulation by treating cells with MG132. We found that lamin B receptor (LBR), a nuclear membrane protein that regulates heterochromatin organization, was mislocalized and down‐regulated in cells on treatment with MG132. Importantly, enforced expression of LBR suppressed cellular senescence induced by MG132. We also showed that LBR was involved in the regulation of chromatin organization in senescent cells, and that endoplasmic reticulum stress and autophagy were likely to be involved in the mislocalization and down‐regulation of LBR. These findings indicate that decreased LBR function was responsible for the induction of cellular senescence by MG132, and thus suggest that protein accumulation caused by inhibition of the proteasome induced cellular senescence probably through chromatin dysregulation in human cells.

## Graphical abstract

Decreased proteasome activity causes protein accumulation that leads to endoplasmic reticulum (ER) stress because of an accumulation of misfolded proteins in ER. Lamin B receptor, a nuclear membrane protein that regulates heterochromatin organization, is misfolded in ER and targeted for degradation by autophagy. Then protein accumulation induces cellular senescence through dysregulation of chromatin caused by decreased lamin B receptor function.

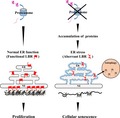

**Abbreviations**
CBB: Coomassie Brilliant Blue
CHX: cycloheximide
DMEM: Dulbecco’s modified Eagle’s medium
Dox: doxycycline
ER: endoplasmic reticulum
LBR: lamin B receptor
SA‐β‐gal: senescence‐associated β‐galactosidase
SAHF: senescence‐associated heterochromatin foci

Normal somatic cells have finite division potential and cease to divide after a limited number of cell divisions, a phenomenon termed replicative senescence 1. Senescent cells become enlarged with an increased protein content and show staining with senescence‐associated β‐galactosidase (SA‐β‐gal) and up‐regulation of senescence‐associated genes 2. They also show a similar phenomenon, termed premature or induced senescence, when they are treated with various types of stress, such as DNA damage, oxidative stress and chromatin destabilization 2. The genes such as *p53*, *p21*, *p16* and *RB* play important roles in cellular senescence of normal types of cell; however, cancer cells that lack these genes still retain the ability to enter cellular senescence on treatment with the stresses 2, 3. These observations indicate that the basic molecular mechanisms for the induction of cellular senescence by the stresses are conserved in normal and cancer cells.

We have analyzed the mechanisms for cellular senescence induced by the stresses in mammalian cells, and found the role of an accumulation of proteins in the induction of cellular senescence 4, 5. Mammalian cells, when treated with the stresses, slow down DNA synthesis but continue protein synthesis, and undergo the phenomenon termed unbalanced growth, which is characterized by an accumulation of proteins 6, 7. Importantly, we have shown that prolonged unbalanced growth leads to cellular senescence 4, 5, 8, and mild restriction of protein synthesis with a low dose of cycloheximide (CHX) 9 suppresses protein accumulation and consequently prevents the induction of cellular senescence by the stresses 10, 11. Moreover, mild restriction of protein synthesis extends not only the replicative life span of normal primary human fibroblasts but also the life span of the nematode *Caenorhabditis elegans* 10, 11. These results suggest that protein accumulation has a causal role in cellular senescence; however, attention has not focused on the role of protein accumulation in cellular senescence.

Coordinated regulation of protein synthesis and degradation is important for the maintenance of protein homeostasis (proteostasis) in cells. Because the proteasome is responsible for major cellular proteolytic activity, inhibition of the proteasome would likely cause protein accumulation (loss of proteostasis). Notably, we have previously shown that inhibition of the proteasome induces cellular senescence in normal primary human fibroblasts, TIG‐7 cells 12, and similar results were obtained by other groups in primary cultured cells 13, 14, 15. Further, the proteolytic activity of the proteasome is decreased in cellular senescence and organismal aging 16. Therefore, these observations are consistent with the view that protein accumulation caused by the decreased proteasome activity may have a role in cellular senescence.

In addition to protein accumulation, a body of evidence indicates that dysregulation of chromatin has a role in cellular senescence 17. Consistent with this, we have previously found that lamin B receptor (LBR), a nuclear membrane protein that regulates heterochromatin organization 18, shows aberrant localization and down‐regulation in senescent cells 19, 20. In addition, we showed that knockdown of *LBR* induces cellular senescence in TIG‐7 cells 20. Thus, decreased LBR function would be causally involved in cellular senescence. Given the role of LBR in heterochromatin organization 18, these findings imply that LBR may regulate cellular senescence through the organization of chromatin.

In this study, we investigated the mechanisms of cellular senescence induced by protein accumulation by treating cells with proteasome inhibitors. We found that protein accumulation caused by the proteasome inhibitors effectively induced cellular senescence with down‐regulation of LBR function. Because enforced expression of LBR suppressed the induction of cellular senescence, down‐regulation of LBR function was responsible for it. In addition, LBR was shown to be involved in the regulation of chromatin organization in senescent cells. Thus, our findings suggested that protein accumulation induced cellular senescence probably through dysregulation of chromatin.

## Materials and methods

### Cell culture

Normal primary human fibroblast TIG‐7 cells and human cervical cancer HeLa cells were purchased from the Japanese Collection of Research Bioresources (Osaka, Japan). TIG‐7 cells were cultured in Dulbecco’s modified Eagle’s medium (DMEM) (Nissui, Tokyo, Japan) supplemented with 10% bovine serum (HyClone, Tokyo, Japan) on tissue culture dishes (Thermo Fisher Scientific, Waltham, MA, USA) under 5% CO_2_ and 95% humidity. Similarly, HeLa cells were cultured in DMEM supplemented with 5% bovine serum, and Hrt7 cells, a HeLa cell line that expresses the reverse tetracycline transactivator, were cultured in DMEM supplemented with 7% bovine serum and 0.4% glucose 21. Cellular senescence was induced by culturing cells with MG132 (Cayman Chemical, Ann Arbor, MN, USA). The dose of MG132 was adjusted based on the cell density because a slightly higher dose of MG132 was required for the effective induction of cellular senescence when cells were plated at a high cell density to prepare protein or RNA samples: 100 nm of MG132 was used for the cells plated at a low cell density (e.g., <5 × 10^3^ cells/35‐mm dish), and 135 nm of it was used for those plated at a high cell density (e.g., >2 × 10^5^ cells/100‐mm dish).

### Colony formation assay

To determine the proliferative potential of cells, we plated appropriate numbers of cells (1.5–5 × 10^3^ cells) on 35‐mm dishes and grew them for 1–2 weeks. The colonies were visualized by staining with Coomassie Brilliant Blue (CBB; Bio‐Rad, Hercules, CA, USA).

### Antibodies

The antibodies against lamin A/C, lamin B, LBR, β‐actin, H4K20me2 and γ‐H2AX were purchased from Santa Cruz Biotechnology (Dallas, TX, USA), Matrix Technology (Maumee, OH, USA), Cosmo Bio (Tokyo, Japan), Wako (Osaka, Japan), Medical & Biological Laboratories (Aichi, Japan) and Cell Signaling (Danvers, MA, USA), respectively.

### Indirect immunofluorescence analysis

Cells were cultured on a coverslip and fixed with methanol for 15 min at −20° C. The cells were incubated with BSA (1%) at room temperature for 1 h and incubated with the primary antibody against LBR, H4K20me2 or γ‐H2AX for 16–24 h. Subsequently, the cells were incubated with an Alexa 568‐conjugated or Alexa 546‐conjugated secondary antibody (Molecular Probes, Eugene, OR, USA) for 3 h, with 4′,6‐diamidino‐2‐phenylindole (DAPI) for 30 min, and mounted with an antifading reagent (Molecular Probes). Fluorescence images were captured by fluorescence microscopy (BZ‐9000; Keyence, Osaka, Japan).

### Western blot analysis

Cells were suspended in the radioimmunoprecipitation assay buffer (20 mm Tris‐HCl, 150 mm NaCl, 1% Nonidet P‐40, 0.5% sodium deoxycholate, 0.1% SDS, 1 mm PMSF, 2 μg·mL^−1^ leupeptin, 2 μg·mL^−1^ aprotinin, 10 mm DTT) and disrupted by sonication for 10–15 s on ice. The cell lysate was subjected to western blot analysis as previously described 3. An ECL chemiluminescence detection kit (GE Healthcare Life Sciences, Tokyo, Japan) and a chemiluminescence image analyzer (ChemiDoc MP System; Bio‐Rad) were used to detect signals.

### Determination of protein content per cell

Protein content in the cell extract was determined with Protein Assay Kit (Bio‐Rad) or Pierce™ (Waltham, MA, USA) BCA Protein Assay Kit (Thermo Fisher Scientific) and normalized by cell number.

### Senescence‐associated β‐galactosidase assay

Cells were fixed with a fixation solution (2% formaldehyde and 0.2% glutaraldehyde) at room temperature for 5 min and incubated with a staining solution (40 mm citric acid–sodium phosphate (pH 6.0), 150 mm NaCl, 2 mm MgC1_2_, 1 mg·mL^−1^ of 5‐bromo‐4‐chloro‐3‐indolyl ß‐d‐galactoside, 5 mm potassium ferricyanide and 5 mm potassium ferrocyanide) at 37° C. After washing with PBS, the cells were photographed under a microscope, and the cells stained blue were counted with imagej software (National Institutes of Health, Bethesda, MD, USA; >150 cells).

### Construction of *LBR* expression plasmids

The *LBR* sequence was cut out from the plasmid carrying LBR cDNA 19 and inserted into pcDNA3.1(−) (Invitrogen, Carlsbad, CA, USA) at the *Eco*RV site to make the plasmid termed pcDNA3.1(−)‐LBR, which expresses *LBR* from the human CMV promoter. The *LBR* sequence [the *Xba*I‐*Eco*RI fragment of pcDNA3.1(−)‐LBR] was cloned into pUHD10‐3 (H. Bujard, University of Heidelberg) to make the plasmid termed pUHD‐LBR, which expresses *LBR* in a doxycycline (Dox)‐dependent manner. To remove the putative motif for chaperone‐mediated autophagy from the LBR sequence, QADIK (103–107) was replaced with QADAA by amplification of the N‐terminal LBR sequence by PCR with the primers 5′‐TGGTCGACCACCTAAAAGTGCCCGCCGATCTGCTTCTGCTTCCCACCAGGCCGACGCTGCAGAAGCAAGG‐3′ and 5′‐GCGGATCCTCGTAGATGTATGGAAATATACG‐3′, and subsequent cloning of the amplified sequence into pCMV‐LBR‐GFP 19 after digestion with *Sal*I and *Bam*HI. The resulting plasmid, pCMV‐LBR(QADAA)‐GFP, expresses the altered LBR protein fused with GFP from the human CMV promoter. GFP was removed from this plasmid by digestion with *Bam*HI and *Not*I, and after blunting and self‐ligation to yield the plasmid, pCMV‐LBR(QADAA).

### Establishment of a cell line that expresses LBR in a Dox‐dependent manner

Hrt7 cells were transfected with pUHD‐LBR, together with pGKpuro, and cultured with puromycin (0.5–1 μg·mL^−1^) for 2 weeks. Approximately 20 colonies were isolated and examined for the Dox‐dependent expression of LBR by western blot analysis.

### DNA transfection

Cells (10^6^) were electroporated with plasmids (10–20 μg) on an electroporator (type NEPA21; Nepa Gene, Chiba, Japan) and were plated and grown on coverslips or tissue culture dishes.

### Quantitative real‐time RT‐PCR

RNA was prepared from cells by the acid guanidinium thiocyanate–phenol–chloroform extraction method 22, and cDNA was synthesized by a reverse transcriptase (PrimeScript first‐strand cDNA synthesis kit; Takara, Shiga, Japan). The transcript of the gene was quantified by quantitative real‐time PCR with a kit (Thunderbird SYBR qPCR Mix; Toyobo, Osaka, Japan) on a StepOnePlus Real‐Time PCR System (Applied Biosystems, Foster City, CA, USA) according to the supplier’s manuals. The primers used were as follows: 5′‐CAGAGGTGGAGAGAGCCAGATT‐3′ and 5′‐CTGGTCCACGGCTCCTTTC‐3′ for *PAI‐1*; 5′‐ACTGAGAGTGATTGAGAGTGGAC‐3′ and 5′‐AACCCTCTGCACCCAGTTTTC‐3′ for *IL‐8*; 5′‐CCAGGCCCAGGTATTGGAGGGG‐3′ and 5′‐GGCCGAGTTCATGAGCCGCA‐3′ for *MMP1*; 5′‐GCTGATTCTGAAGCCATTTG‐3′ and 5′‐CTGTGTTGCTATGTAACTGC‐3′ for *LBR*; 5′‐TGCTGAGTCCGCAGCAGGTG‐3′ and 5′‐GCTGGCAGGCTCTGGGGAAG‐3′ for *XBP1*; 5′‐CTGGGTACATTTGATCTGACTGG‐3′ and 5′‐GCATCCTGGTGGCTTTCCAGCCAT TC‐3′ for *GRP78*; and 5′‐GAAGGTGAAGGTCGGAGTCAA‐3′ and 5′‐GACAAGCTTCCCGTTCTCAG‐3′ for *GAPDH*. The expression level of each gene was normalized with the expression of *GAPDH*.

### Statistical analysis

Data are expressed as mean ± standard deviation. Statistical significance was determined by a Student’s *t*‐test or one‐way ANOVA followed by Tukey–Kramer test.

## Results

### Induction of cellular senescence by MG132 in HeLa cells

**Figure 1:**
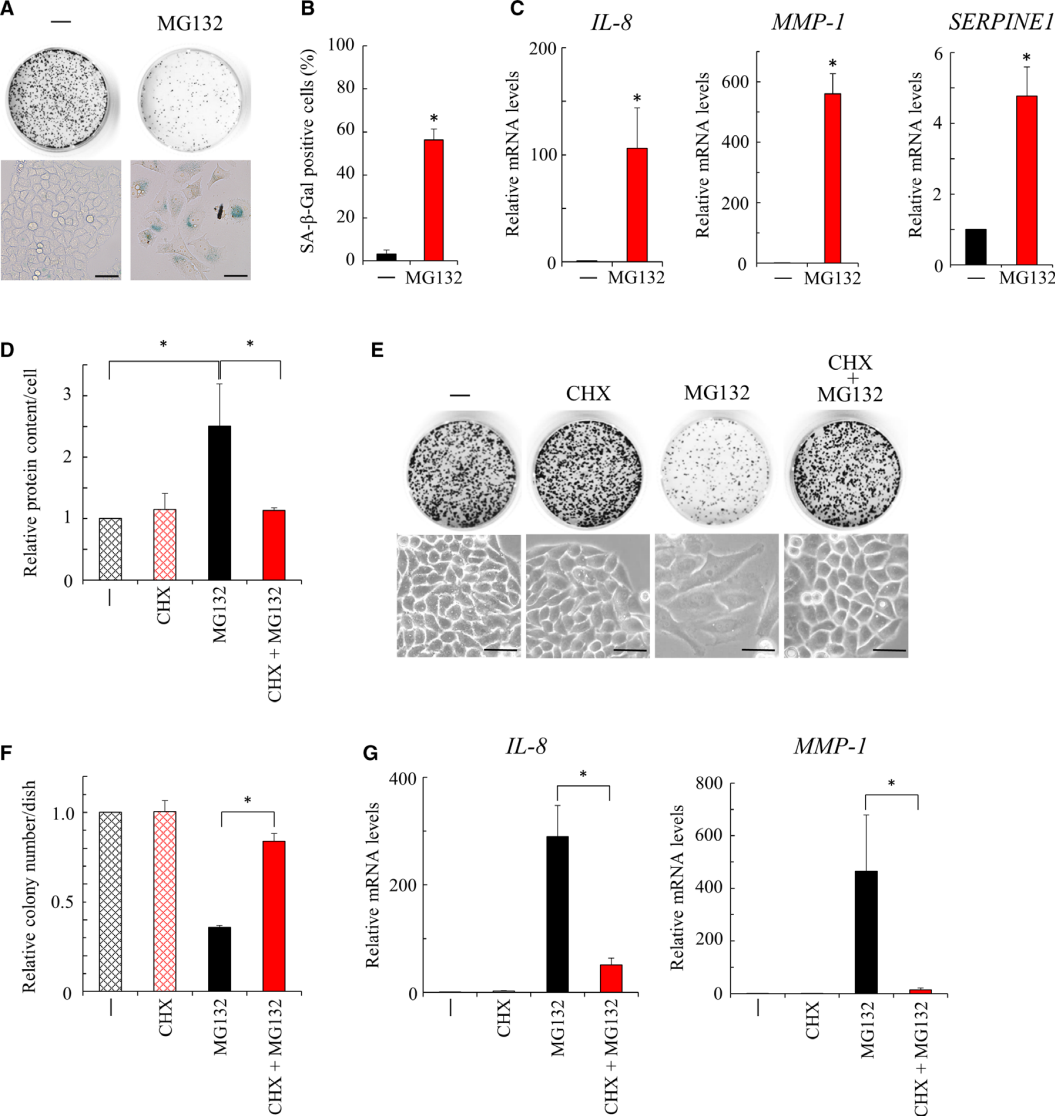
Cellular senescence induced by MG132. (A) HeLa cells were treated with MG132 (100 nm) for 5 days and stained with SA‐β‐gal (lower). Formed colonies were stained with CBB (upper). Scale bars: 50 μm. (B) The percentage of the SA‐ß‐gal‐positive cells (A) was determined (>150 cells, *n* = 3). An asterisk indicates statistical significance, *P < 0.05 (Student’s *t*‐test). Error bars indicate SD. (C) Expression of the senescence‐associated genes (*IL‐8*, *MMP‐1* and *SERPINE1*) was determined by quantitative RT–PCR analysis in HeLa cells treated with MG132 (135 nm) for 4 days (*n* = 3). An asterisk indicates statistical significance, *P < 0.05 (Student’s *t*‐test). Error bars indicate SD. (D) Protein content per cell was determined in HeLa cells treated with MG132 (135 nm) in the presence or absence of CHX (0.04 μg·mL^−1^). Protein content is expressed as a value relative to that of the cells untreated with MG132 or CHX (*n* = 4). An asterisk indicates statistical significance, *P < 0.05 (one‐way ANOVA and Tukey–Kramer test). Error bars indicate SD. (E) HeLa cells were treated with MG132 (100 nm) in the presence or absence of CHX (0.04 μg·mL^−1^) for 4 days, and cell morphology was photographed (lower). Cells were subsequently cultured for 7 days after replating to new dishes (1500 cells/35‐mm dish) to observe their proliferative potential, and formed colonies were stained with CBB (upper). Scale bars: 50 μm. (F) The number of colonies (E) is expressed as a value relative to that of the cells not treated with MG132 or CHX (*n* = 3). An asterisk indicates statistical significance, *P < 0.05 (one‐way ANOVA and Tukey–Kramer test). Error bars indicate SD. (G) Expression of the senescence‐associated genes (*IL‐8* and *MMP‐1*) was determined by quantitative RT–PCR analysis in HeLa cells treated with MG132 (135 nm) in the presence or absence of CHX (0.04 μg·mL^−1^) for 4 days (*n* = 3). An asterisk indicates statistical significance, *P < 0.05 (one‐way ANOVA and Tukey–Kramer test). Error bars indicate SD.

We have previously shown that MG132, an inhibitor of the proteasome 23, induced cellular senescence in normal primary human fibroblast TIG‐7 cells 12; then we examined whether a similar phenomenon is observed in cancerous HeLa cells. We found that HeLa cells, when treated with MG132, ceased to divide with enlarged and flattened cell morphology, and showed decreased colony formation (Fig. 1A). Further, these cells were stained with SA‐β‐gal, a typical marker of senescent cells (Fig. 1A,B), and up‐regulated the expression of the senescence‐associated genes, such as *IL‐8* (interleukin‐8), *MMP‐1* (collagenase) and *SERPINE1* (*PAI‐1*) (Fig. 1C). Of note, similar senescence‐like morphology was observed in HeLa cells treated with bortezomib, another type of proteasome inhibitor (Fig. S1). These observations suggested that MG132 induced cellular senescence in HeLa cells, as well as in TIG‐7 cells 12; thus, the mechanisms for the induction of cellular senescence by MG132 can be analyzed in both HeLa and TIG‐7 cells.

### Role of protein accumulation in MG132‐induced cellular senescence

We measured the protein contents of MG132‐treated HeLa and TIG‐7 cells, and found that these cells showed an increase in protein content per cell, a typical feature of senescent cells (Figs 1D and S2). We then examined the effect of suppression of protein accumulation on the induction of cellular senescence. For this, we suppressed protein accumulation by restricting protein synthesis with a low concentration of CHX (0.04 μg·mL^−1^), an inhibitor of protein synthesis, and found that CHX at this concentration slightly slowed cell growth (Fig. 1E), but effectively suppressed the protein accumulation induced by MG132 (Fig. 1D). Importantly, we found that the cells proliferated with almost normal cell morphology when treated with MG132 in the presence of CHX, although they entered cellular senescence with enlarged and flattened cell morphology when treated with MG132 alone (Fig. 1E,F). Further, CHX suppressed the elevated expression of the senescence‐associated genes in the cells treated with MG132 (Fig. 1G). These findings indicated that suppression of protein accumulation by CHX prevented the induction of cellular senescence by MG132, and thus suggested that protein accumulation (loss of proteostasis) was causally involved in the induction of cellular senescence by MG132.

### Role of DNA damage in MG132‐induced cellular senescence

We also examined the implication of DNA damage in the induction of cellular senescence by MG132 because DNA damage is well‐known to be involved in cellular senescence. However, we did not observe the induction of γ‐H2AX foci formation, a hallmark of DNA damage, in the cells treated with MG132 (Fig. S3). Also, DNA damage did not appear to be involved in the induction of cellular senescence by MG132.

### Aberrant localization and down‐regulation of LBR in MG132‐induced cellular senescence

**Figure 2:**
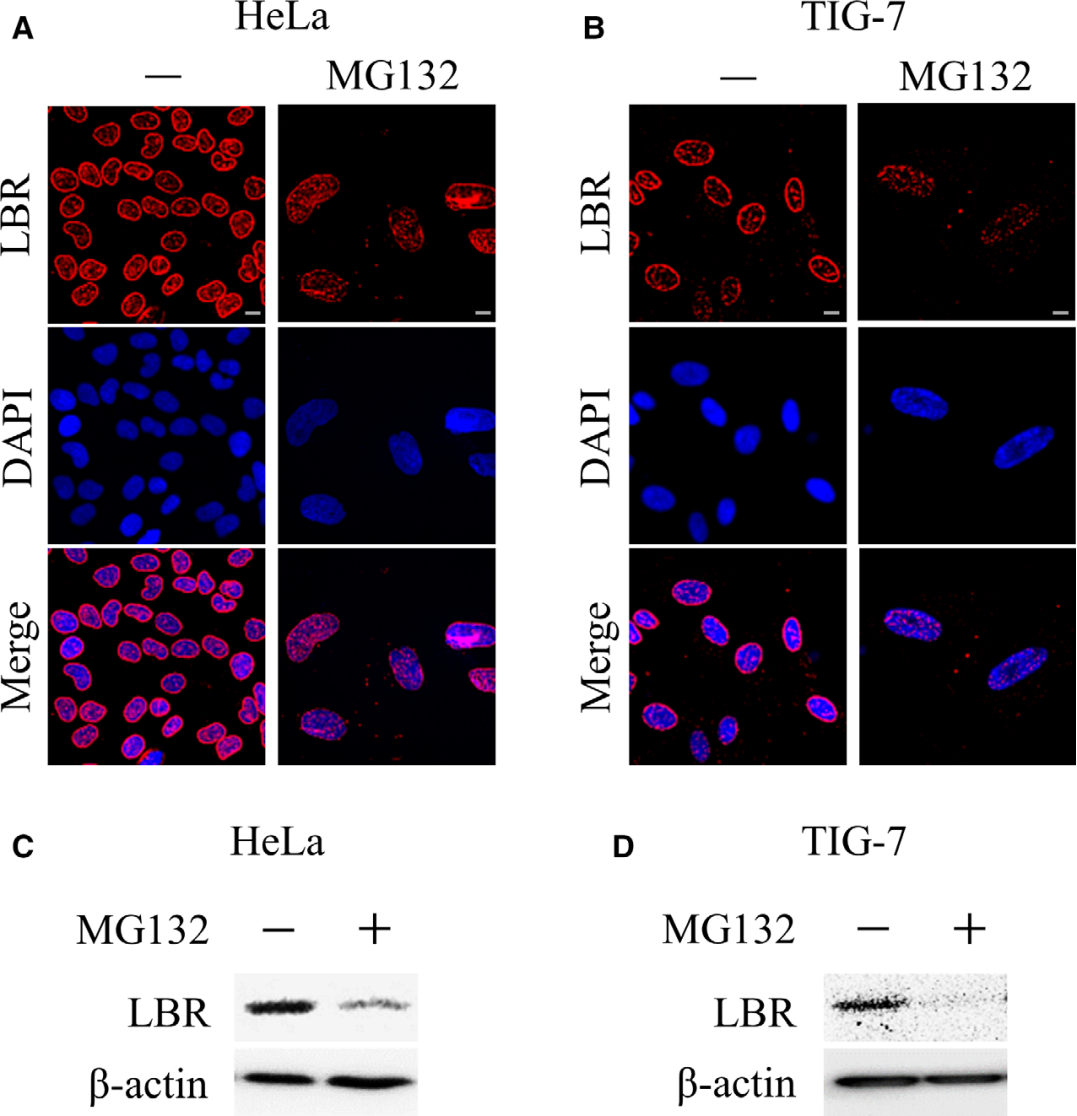
LBR in MG132‐induced senescent cells. (A, B) Localization of LBR was determined by immunohistochemical analysis with an antibody against LBR in HeLa cells (A) and TIG‐7 cells (36 PDLs) (B) treated with MG132 (100 nm) for 4 days. DNA was stained with DAPI. Scale bars: 10 μm. (C, D) Protein lysates were prepared from HeLa cells (C) and TIG‐7 cells (D) treated with MG132 (135 nm) for 4 days and then subjected to western blot analysis with an antibody against LBR.

We have previously shown that mislocalization and down‐regulation of LBR are observed in BrdU‐induced, excess thymidine‐induced, and replicative senescent cells 19, 20. We then examined the localization of LBR in the cells treated with MG132, and found that they showed a similar change in the localization of LBR; that is, proliferating cells showed a normal nuclear peripheral localization pattern of LBR, but MG132‐treated cells showed a diffusive distribution pattern of it (Fig. 2A,B). In addition, western blot analysis revealed that the LBR protein was down‐regulated on treatment with MG132 by 40% and 70% in HeLa and TIG‐7 cells, respectively (Fig. 2C,D). Thus, mislocalization and down‐regulation of LBR were observed in cellular senescence induced by MG132 as well.

### Implication of LBR in MG132‐induced cellular senescence

**Figure 3:**
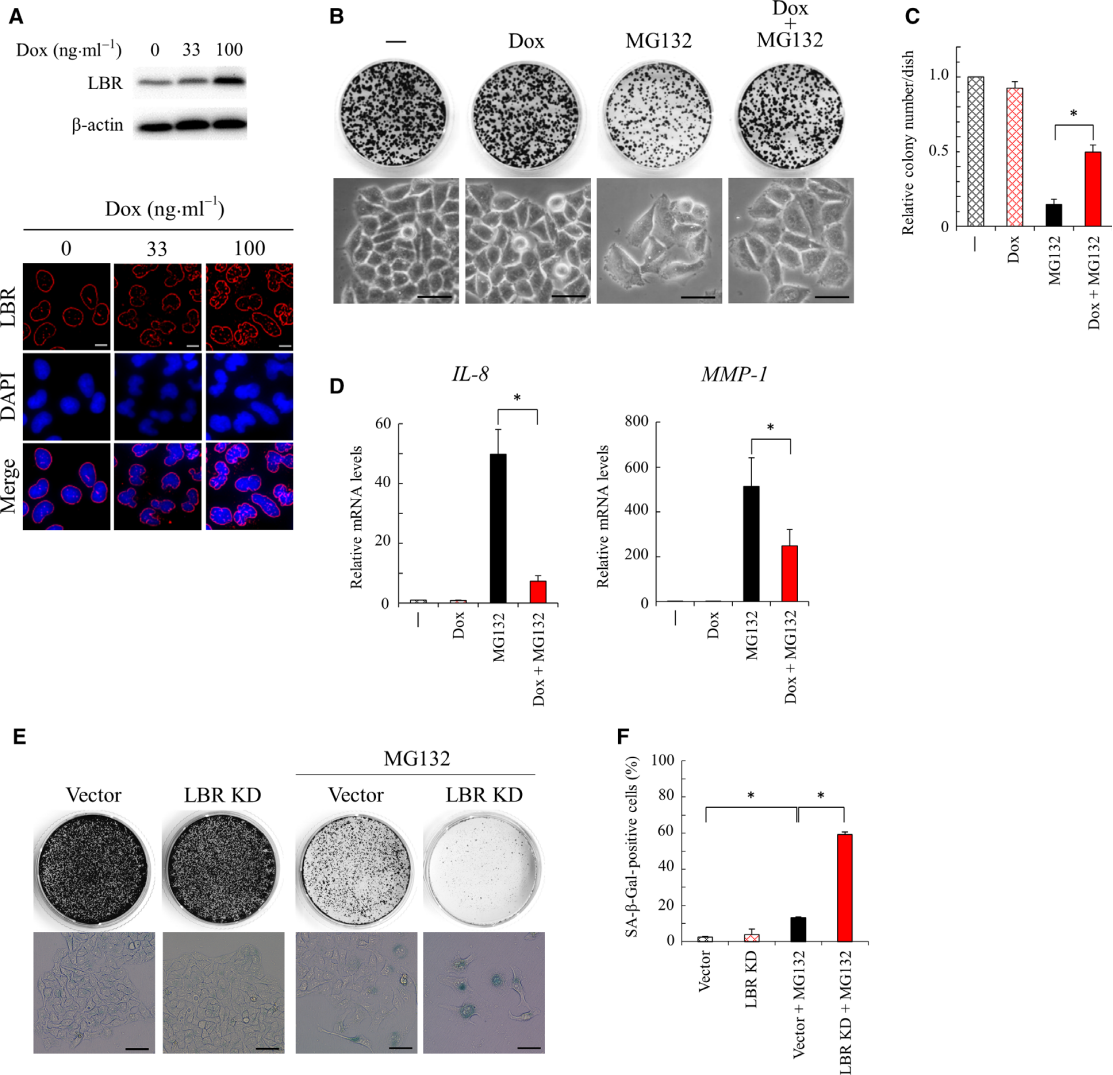
Roles of *LBR* in the induction of cellular senescence in HeLa cells. (A) Protein lysates were prepared from HeLa^T‐LBR^ cells treated with Dox (0–100 ng·m^−1^) for 4 days and subjected to western blot analysis with an antibody against LBR (upper). Localization of LBR was determined by immunohistochemical analysis with an antibody against LBR in HeLa^T‐LBR^ cells treated with Dox (0–100 ng·mL^−1^) for 3 days. DNA was stained with DAPI. Scale bars: 10 μm. (B) HeLa^T‐LBR^ cells were treated with MG132 (100 nm) in the presence or absence of Dox (100 ng·mL^−1^) for 4 days, and cell morphology was photographed (lower). Cells were subsequently cultured for 12 days after replating to new dishes (1500 cells/35‐mm dish) to observe their proliferative potential, and formed colonies were stained with CBB (upper). Scale bars: 50 μm. (C) The number of colonies (B) is expressed as a value relative to that of the cells untreated with MG132 or Dox (*n* = 3). An asterisk indicates statistical significance, *P < 0.05 (one‐way ANOVA and Tukey–Kramer test). Error bars indicate SD. (D) Expression of the senescence‐associated genes (*IL‐8* and *MMP‐1*) was determined by quantitative RT–PCR analysis in HeLa cells treated with MG132 (135 nm) in the presence or absence of Dox (100 ng·mL^−1^) for 4 days. An asterisk indicates statistical significance, *P < 0.05 (one‐way ANOVA and Tukey–Kramer test). Error bars indicate SD. (E) HeLa cells were transfected with the LBR knockdown vector or an empty vector and treated with MG132 (100 nm) for 3 days, and subsequently cultured with fresh medium for 2 days. Cells were stained with SA‐β‐gal (lower), and formed colonies were stained with CBB (upper). Scale bars: 50 μm. (F) The percentage of the SA‐ß‐gal‐positive cells (A) was determined (>150 cells, *n* = 3). An asterisk indicates statistical significance, *P < 0.05 (one‐way ANOVA and Tukey–Kramer test). Error bars indicate SD.

Because the earlier findings suggested the implication of decreased LBR function in cellular senescence, we examined whether enforced expression of *LBR* suppresses the induction of cellular senescence by MG132. To express *LBR* in a regulated manner, we used a Tet‐ON system in HeLa cells and successfully isolated a cell line, termed HeLa^T‐LBR^, which increased the expression of LBR in a Dox‐dependent manner (Fig. 3A). HeLa^T‐LBR^ cells were treated with less than 100 ng·mL^−1^ Dox because they showed abnormal nuclear morphology when treated with a greater dose of Dox (Fig. S4). We then examined the effect of enforced expression of *LBR* on the induction of cellular senescence. HeLa^T‐LBR^ cells treated with MG132 alone entered cellular senescence and consequently showed decreased colony formation after replating; however, HeLa^T‐LBR^ cells treated with MG132 in the presence of Dox did not enter cellular senescence, and thus showed improved colony formation after replating (Fig. 3B,C). Similar results were obtained in HeLa^T‐LBR^ cells treated with bortezomib in the presence of Dox (Fig. S5). Furthermore, Dox significantly suppressed the elevated expression of the senescence‐associated genes in the cells treated with MG132 (Fig. 3D). These findings indicated that enforced expression of *LBR* suppressed the induction of cellular senescence by MG132 in HeLa cells. In addition, because enforced expression of *LBR* also suppressed it in TIG‐7 cells (Fig. 5G), the role of LBR in MG132‐induced cellular senescence would be conserved in HeLa and TIG‐7 cells.

We next determined the effect of decreased expression of *LBR* on the induction of cellular senescence by MG132. We down‐regulated the expression of *LBR* by transfecting the knockdown vector that expresses an shRNA against *LBR* 20. Knockdown of *LBR* induces cellular senescence in TIG‐7 cells 20, but not in HeLa cells (Fig. 3E,F). We then examined the effect of knockdown of *LBR* on the induction of cellular senescence by MG132 in HeLa cells. HeLa cells were weakly induced to undergo cellular senescence when treated with MG132 for 3 days (~15% of the cells), compared with for 5 days (Figs 1A,B and 3E,F). However, importantly, knockdown of *LBR* greatly increased the population of the senescent cells in HeLa cells treated with MG132 for 3 days (~60% of the cells) (Fig. 3E,F). This observation indicated that knockdown of *LBR* facilitated the induction of cellular senescence by MG132 in HeLa cells. Then MG132‐induced cellular senescence was suppressed by enforced expression of *LBR* and facilitated by knockdown of *LBR*. These results suggested that decreased LBR function was crucially involved in the induction of cellular senescence by MG132.

### Relevance between protein accumulation and LBR in MG132‐induced cellular senescence

**Figure 4:**
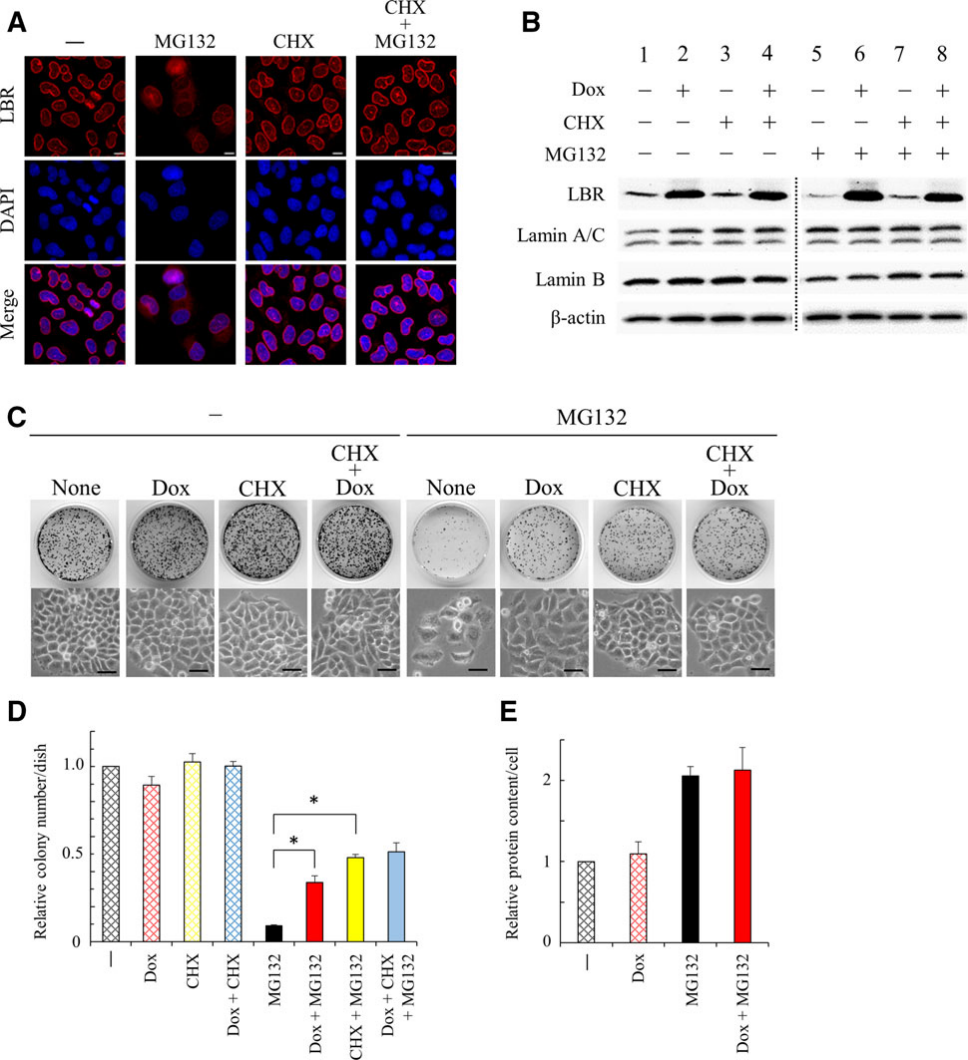
Relevance between protein accumulation and LBR. (A) Localization of LBR was determined by immunohistochemical analysis with an antibody against LBR in HeLa cells treated with MG132 (100 nm) in the presence or absence of CHX (0.04 μg·mL^−1^) for 4 days. DNA was stained with DAPI. Scale bars: 10 μm. (B) Protein lysates were prepared from HeLa^T‐LBR^ cells treated with MG132 (135 nm) ± CHX (0.04 μg·mL^−1^) ± Dox (100 ng·mL^−1^) for 4 days, and were subjected to western blot analysis with antibodies against LBR and lamins. (C) HeLa^T‐LBR^ cells were treated with MG132 (100 nm) ± CHX (0.04 μg·mL^−1^) ± Dox (100 ng·mL^−1^) for 4 days, and cell morphology was photographed (lower). The cells were subsequently cultured for 8 days after replating to new dishes (1500 cells/35‐mm dish) to observe their proliferative potential, and formed colonies were stained with CBB (upper). Scale bars: 50 μm. (D) The number of colonies (C) is expressed as a value relative to that of the cells untreated with MG132, CHX or Dox (*n* = 3). An asterisk indicates statistical significance, *P < 0.05 (one‐way ANOVA and Tukey–Kramer test). Error bars indicate SD. (E) Protein content was determined in HeLa^T‐LBR^ cells treated with MG132 (135 nm) in the presence or absence of Dox (100 ng·mL^−1^) for 4 days (*n* = 3, one‐way ANOVA and Tukey–Kramer test). Error bars indicate SD.

The earlier findings suggested the involvement of both protein accumulation and decreased LBR function in the induction of cellular senescence by MG132. To understand the relevance between protein accumulation and LBR, we examined the effect of suppression of protein accumulation on MG132‐induced changes in LBR. We then found that CHX, which suppresses protein accumulation (Fig. 1D), prevented not only the mislocalization but also the down‐regulation of LBR induced by MG132 (Fig. 4A,B, lane 1 versus lane 5 versus lane 7). These findings suggested that protein accumulation led to mislocalization and down‐regulation of LBR. We also examined the combined effect of CHX and Dox on MG132‐induced cellular senescence. We found that, even though Dox or CHX alone showed a suppressive effect on MG132‐induced cellular senescence (Figs 1E, 3B and 4C), a combination of Dox and CHX did not show an additive suppressive effect on it (Fig. 4C,D). These findings suggested that protein accumulation and down‐regulation of LBR appear to lie in the same genetic pathway of MG132‐induced cellular senescence, and protein accumulation would probably locate upstream of down‐regulation of LBR. This is consistent with the finding that enforced expression of LBR by Dox failed to suppress protein accumulation on treatment with MG132 (Fig. 4E).

### Regulatory mechanisms of LBR in MG132‐induced cellular senescence

**Figure 5:**
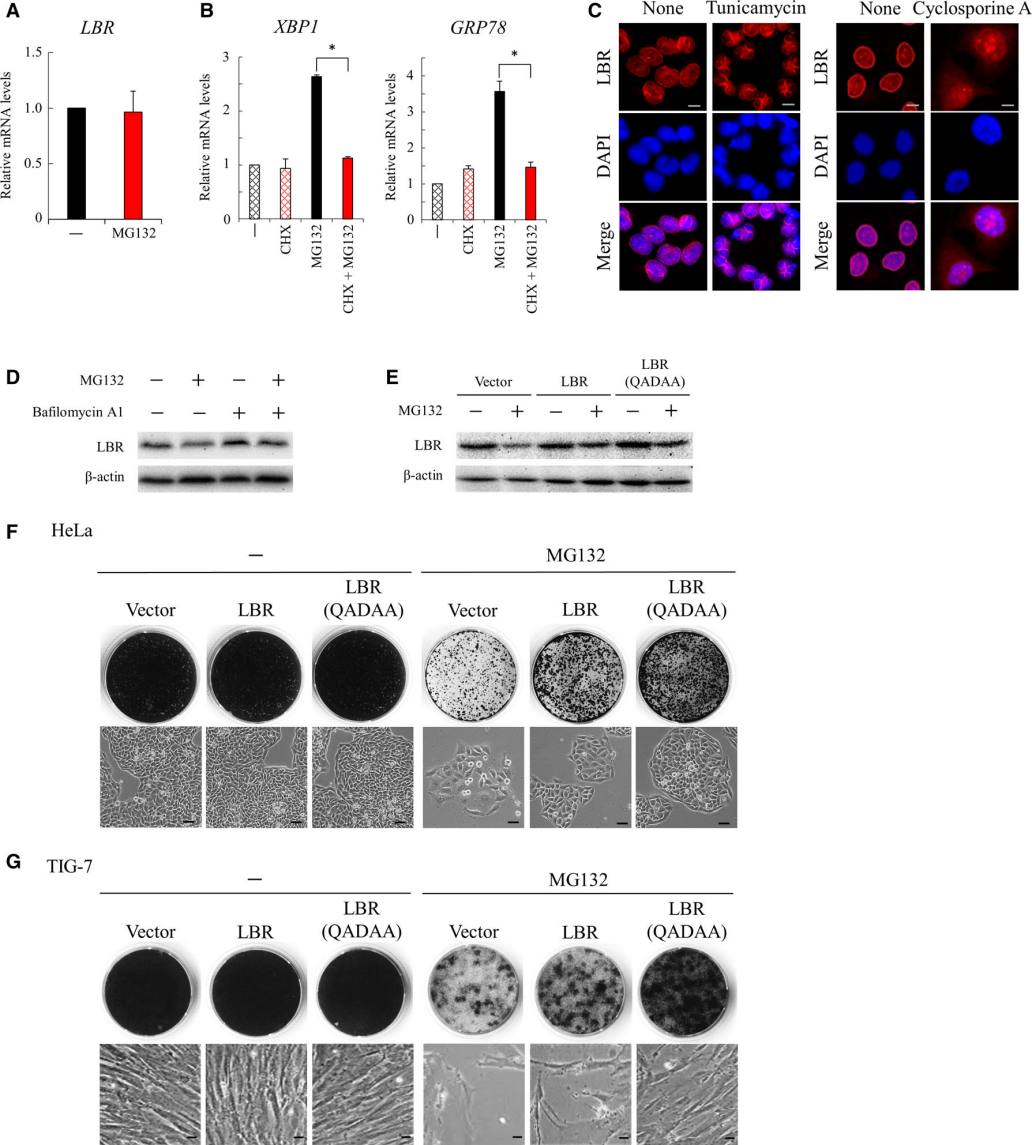
Regulation of LBR by ER stress and autophagy. (A) Expression of LBR was determined by quantitative RT–PCR analysis in HeLa cells treated with MG132 (135 nm) for 4 days (*n* = 3, Student’s *t*‐test). Error bars indicate SD. (B) Expression of the ER stress marker genes (*XBP‐1* and *GRP78*) was determined by quantitative RT–PCR analysis in HeLa cells treated with MG132 (135 nm) in the presence or absence of CHX (0.04 μg·mL^−1^) for 4 days (*n* = 3). An asterisk indicates statistical significance, *P < 0.05 (one‐way ANOVA and Tukey–Kramer test). Error bars indicate SD. (C) Localization of LBR was determined by immunohistochemical analysis with an antibody against LBR in HeLa cells treated with tunicamycin (5 μg·mL^−1^) for 24 h or cyclosporine A (20 μg·mL^−1^) for 7 days. DNA was stained with DAPI. Scale bars: 10 μm. (D) HeLa cells were treated with or without MG132 (135 nm) for 4 days and subsequently cultured in the presence or absence of bafilomycin A1 (100 nm) for 8 h. Protein lysates were prepared from these cells and subjected to western blot analysis with an antibody against LBR. (E) HeLa cells were transfected with an empty vector, pCMV‐LBR or pCMV‐LBR(QADAA), and treated with MG132 for 4 days. Protein lysates were prepared from these cells and subjected to western blot analysis with an antibody against LBR. (F, G) HeLa cells (F) or TIG‐7 cells (G) were transfected with an empty vector, pCMV‐LBR‐GFP or pCMV‐LBR(QADAA)‐GFP, and treated with MG132 for 4 days. These cells were subsequently cultured with fresh medium to observe their proliferative potential. Cell morphology was photographed (lower), and formed colonies were stained with CBB (upper). Scale bars: 50 μm.

We explored the mechanism for the down‐regulation of LBR by MG132. Quantitative RT–PCR analysis indicated that the mRNA level of LBR was not altered by treatment with MG132 (Fig. 5A); then LBR was down‐regulated through post‐transcriptional mechanisms. Importantly, proteasome inhibitors are shown to cause endoplasmic reticulum (ER) stress because of an accumulation of misfolded proteins in the ER and to induce autophagy 24, 25, 26, 27. Because LBR is transferred from the ER to the nuclear membrane 28, we examined the implication of ER stress in MG132‐induced cellular senescence. We found that MG132‐treated cells showed increased expression of *GRP78* and spliced *XBP1*, which indicated that ER stress was induced by MG132 (Fig. 5B). Further, of note, ER stress induced by MG132 was effectively suppressed by CHX (Fig. 5B). We then examined the localization of LBR on induction of ER stress by tunicamycin or cyclosporine A 29, and found that ER stress caused abnormal localization of LBR (Fig. 5C). These findings suggested the possibility that ER stress induced by MG132 may be involved in the mislocalization of LBR.

In addition, we examined the implication of autophagy in the down‐regulation of LBR, and found that its down‐regulation was reduced by half (20% decrease) by addition of bafilomycin A1, which inhibits acidification of the lysosome 30 (Fig. 5D). Then LBR was suggested to be degraded at least partly by the lysosome in MG132‐treated cells. Because LBR possesses a putative motif required for chaperone‐mediated autophagy, QADIK (103–107), we replaced it with QADAA to yield LBR(QADAA), which is likely more resistant to degradation by autophagy 31. When wild‐type LBR or LBR(QADAA) was expressed in cells, LBR(QADAA) was less effectively down‐regulated than wild‐type LBR by treatment with MG132 [10% decrease by LBR(QADAA) compared with 40% decrease by an empty vector] (Fig. 5E). This observation suggested that LBR would be at least partly degraded by autophagy induced by MG132. Consistent with this finding, we observed that expression of LBR(QADAA) more effectively suppressed the induction of cellular senescence by MG132 than that of wild‐type LBR in HeLa cells (Fig. 5F) and also in TIG‐7 cells (Fig. 5G). Taken together, these findings suggested the possibility that ER stress and autophagy that are induced by MG132 would be involved in the mislocalization and down‐regulation of LBR in MG132‐induced senescent cells.

### The role of LBR in senescence‐associated heterochromatin foci formation induced by MG132

**Figure 6:**
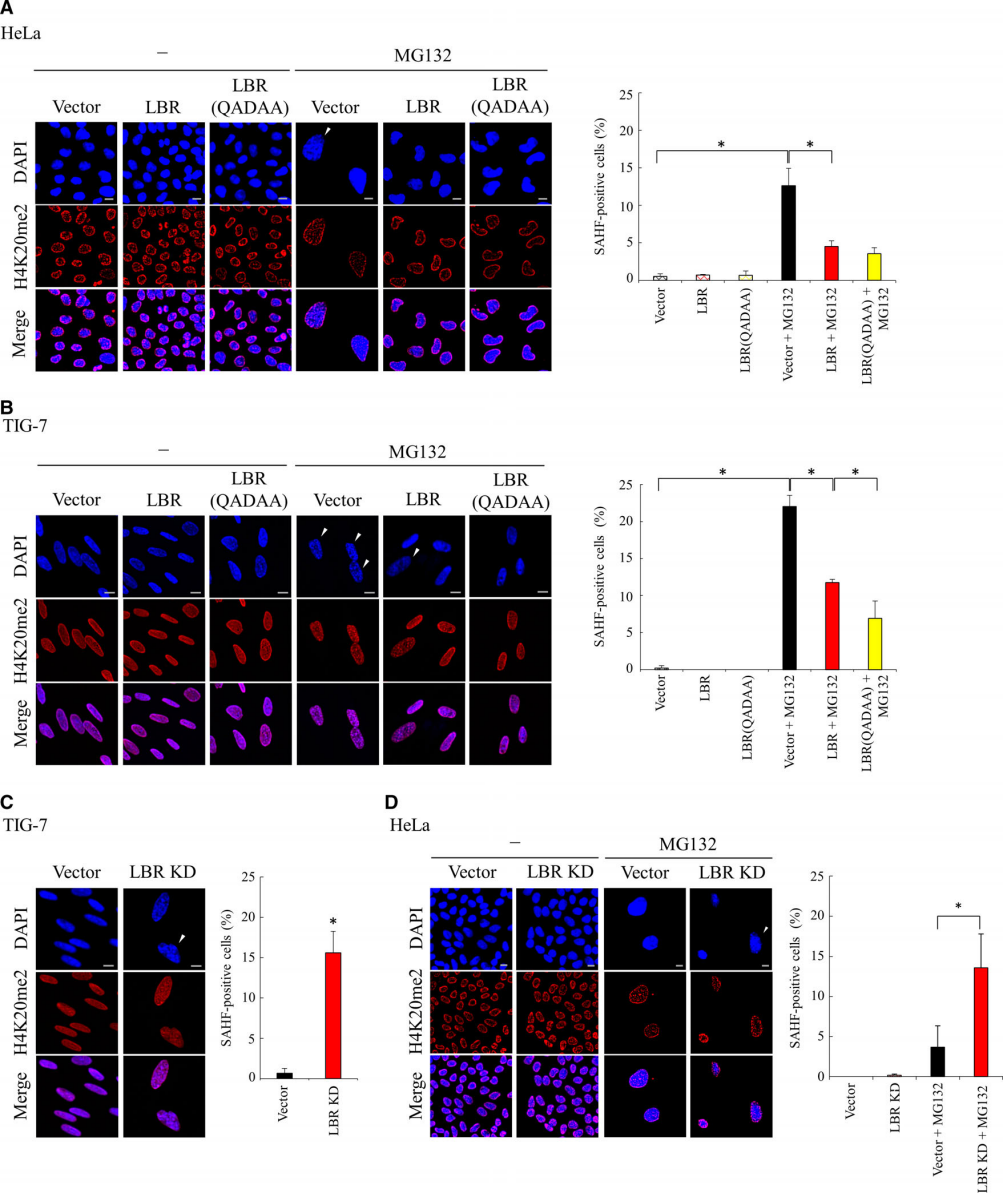
Roles of *LBR* in the chromatin organization. (A, B) HeLa cells (A) or TIG‐7 cells (B) were transfected with an empty vector, pCMV‐LBR or pCMV‐LBR(QADAA), and treated with MG132 for 4 days. DNA and H4K20me2 were stained with DAPI and an antibody against H4K20me2, respectively. Arrowhead indicates the nuclei with SAHF. Scale bars: 10 μm (left). The percentage of the SAHF‐positive cells was determined (>100 cells, *n* = 3; right). An asterisk indicates statistical significance, *P < 0.05 (one‐way ANOVA and Tukey–Kramer test). Error bars indicate SD. (C) TIG‐7 cells were transfected with an empty vector or LBR knockdown vector. DNA and H4K20me2 were stained with DAPI and an antibody against H4K20me2, respectively. Arrowhead indicates the nuclei with SAHF. Scale bars: 10 μm (left). The percentage of the SAHF‐positive cells was determined (>100 cells, *n* = 3; right). An asterisk indicates statistical significance, *P < 0.05 (Student’s *t*‐test). Error bars indicate SD. (D) HeLa cells were transfected with the LBR knockdown vector or an empty vector, and cultured with MG132 for 3 days (a shorter period than 4 days in A). DNA and H4K20me2 were stained as in (C). The percentage of the SAHF‐positive cells was determined (>100 cells, *n* = 3; right). An asterisk indicates statistical significance, *P < 0.05 (one‐way ANOVA and Tukey–Kramer test). Error bars indicate SD.

To investigate the role of LBR in the regulation of chromatin in senescent cells, we examined the effect of expression of LBR on senescence‐associated heterochromatin foci (SAHF) formation induced by MG132 in HeLa and TIG‐7 cells 12. We found that expression of wild‐type LBR or LBR(QADAA) significantly suppressed the induction of SAHF formation by MG132 (Fig. 6A,B). Interestingly, dimethylation of histone H4 at lysine 20 (H4K20me2), which interacts with LBR 32, preferentially localized at the nuclear membrane periphery in proliferating cells; however, it roughly colocalized with SAHF in the nucleoplasm in MG132‐treated cells (Fig. 6A,B). Further, we found that knockdown of *LBR* increased SAHF formation in TIG‐7 cells (Fig. 6C) and facilitated SAHF formation induced by MG132 in HeLa cells (Fig. 6D). These observations suggested a possible role of LBR in the regulation of SAHF formation in senescent cells.

## Discussion

Coordinated regulation of protein synthesis and degradation is important for the maintenance of proteostasis. Mammalian cells slow down DNA synthesis but continue protein synthesis when treated with stresses; then they accumulate proteins and undergo unbalanced growth, which eventually leads to cellular senescence 4, 5, 6, 7, 8, 10. Therefore, protein accumulation (loss of proteostasis) appears to be one of the important factors that induce cellular senescence. To analyze the role of protein accumulation in cellular senescence, we focused on the proteasome. Because the proteasome is responsible for a major cellular proteolytic activity, it is likely that inhibition of the proteasome would cause protein accumulation that leads to cellular senescence. Indeed, we and other groups have previously shown that inhibition of the proteasome induces cellular senescence in primary cultured cells 12, 13, 14, 15, and in this study, we showed a similar phenomenon in cancer cells as well. Therefore, both normal and cancer cells were induced to undergo cellular senescence by treatment with the proteasome inhibitor, MG132, and then the basic mechanisms for MG132‐induced cellular senescence can be analyzed in HeLa cells and TIG‐7 cells.

We showed that protein accumulation caused mislocalization and down‐regulation of LBR. Further, importantly, enforced expression of *LBR* suppressed the induction of cellular senescence by MG132 and, conversely, knockdown of *LBR* facilitated it. Then protein accumulation induced cellular senescence through the down‐regulation of LBR function. Notably, we have also shown the crucial roles of LBR in cellular senescence by the findings that knockdown of *LBR* induces cellular senescence in TIG‐7 cells and facilitates the induction of cellular senescence by excess thymidine in HeLa cells 20. In addition, we explored the regulatory mechanism of LBR and found that ER stress and autophagy that are induced by MG132 were likely involved in the mislocalization and down‐regulation of LBR in senescent cells. Then it is possible to speculate that: (a) the decreased proteasome activity causes protein accumulation that leads to ER stress because of an accumulation of misfolded proteins in ER and the induction of autophagy; (b) the misfolded LBR protein, which is not properly transferred from the ER to the nuclear membrane, is targeted for degradation by autophagy; and then (c) down‐regulated LBR function induces cellular senescence.

How does LBR regulate the induction of cellular senescence? LBR regulates the organization of heterochromatin at the nuclear membrane periphery, probably through the interaction with the chromatin‐associated proteins, such as lamin B, HP1, MeCP2 and H4K20me2 32, 33, 34, 35, 36. Indeed, the absence of LBR and lamin A causes detachment of heterochromatin from the nuclear membrane and redistribution of it in the center of the nucleus in all postmitotic cells 18, and knockdown of *LBR* causes relocalization and distension of centromeric heterochromatin in human cells 37. In this study, we showed that LBR was involved in the regulation of SAHF formation. Therefore, it is likely that LBR regulates cellular senescence through chromatin organization. Because gene expression is spatially regulated through chromatin organization, altered chromatin organization would lead to substantial changes in gene expression. Therefore, it is possible to speculate that altered chromatin organization due to decreased LBR function may result in misexpression of genes and consequently trigger the induction of cellular senescence. In this regard, it is intriguing to speculate that down‐regulation of LBR function may have a role in the altered gene expression observed in senescent cells.

**Figure 7:**
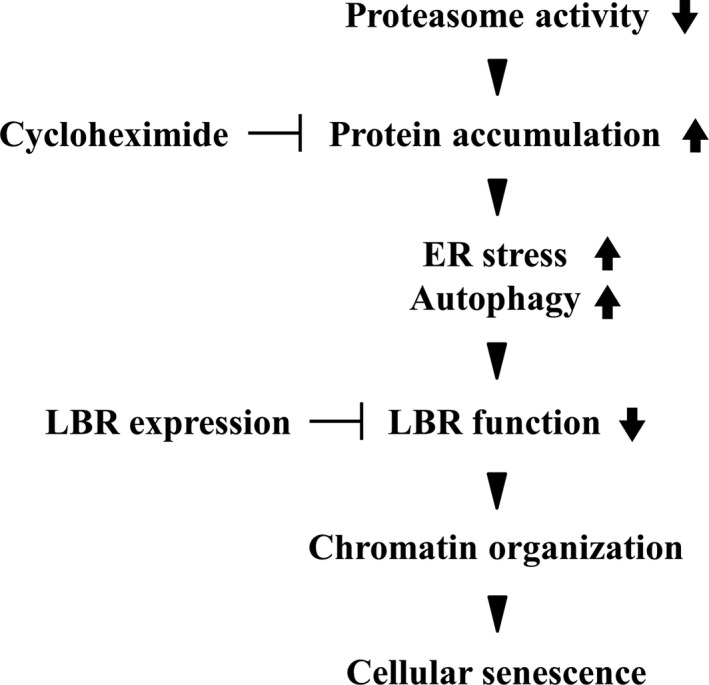
Schematic illustration of the model for cellular senescence induced by proteasome inhibition.

Finally, we present a model of the mechanism for cellular senescence induced by inhibition of the proteasome (Fig. 7). Because a substantial body of evidence indicates the decreased proteasome activity in cellular senescence and organismal aging, our findings obtained in MG132‐induced cellular senescence might be applicable to senescence in various cell types and organisms. Our findings would provide a novel insight into the mechanisms underlying cellular senescence.

## Conflict of interest

The authors declare no conflict of interest.

## Author contributions

AE carried out experiments. YT, KM and DA contributed to data analysis. MF supervised the project.

## Supporting information


**Fig. S1** **.** Induction of cellular senescence by bortezomib in HeLa cells. (A) HeLa cells were treated with bortezomib (3 nm) for 7 days. Cell morphology was photographed (lower), and formed colonies were stained with CBB (upper). Scale bars: 50 μm. (B) The percentage of the SA‐ß‐gal‐positive cells (A) was determined (>150 cells, *n* = 3). An asterisk indicates statistical significance, *P < 0.05 (Student’s *t*‐test). Error bars indicate SD.

**Fig. S2** **.** Protein content in TIG‐7 cells treated with MG132. Protein content per cell was determined in TIG‐7 cells treated with MG132 (135 nm) for 4 days. Protein content is expressed as a value relative to that of the cells not treated with MG132 (*n* = 3). An asterisk indicates statistical significance, *P < 0.05 (Student’s *t*‐test). Error bars indicate SD.

**Fig. S3** **.** DNA damage in the cells treated with MG132. (A) DNA damage was examined by immunostaining HeLa cells treated with MG132 (100 nm) or excess thymidine (EdT, 1.5 mm) for 4 days with an antibody against γ‐H2AX. DNA was stained with DAPI. Excess thymidine was used to induce DNA damage in HeLa cells. Scale bars: 10 μm. (B) The percentage of the cells with γ‐H2AX foci (A) was determined (>50 cells, *n* = 3). An asterisk indicates statistical significance, *P < 0.05 (one‐way ANOVA and Tukey–Kramer test). Error bars indicate SD.

**Fig. S4** **.** Abnormal nuclear morphology induced by a high dose of Dox. Nuclear membrane was stained with an antibody against LBR in HeLa^T‐LBR^ cells treated with Dox (1000 ng·mL^−1^) for 7 days. DNA was stained with DAPI. Scale bars: 10 μm.

**Fig. S5** **.** Effect of enforced expression of LBR on the induction of cellular senescence by bortezomib in HeLa cells. HeLa^T‐LBR^ cells were treated with bortezomib (3 nm) in the presence or absence of Dox (33 ng·mL^−1^) for 4 days, and cell morphology was photographed (lower). Cells were cultured for 9 days after replating to new dishes and then stained with CBB (upper). Scale bars: 50 μm.
