# Supplementary figures and images for: Lamin B receptor plays a key role in cellular senescence induced by inhibition of the proteasome
Source: FEBS Open Bio. 2020 Jan 6;10(2):237–50. doi: 10.1002/2211-5463.12775 (PMC6996348; doi:10.1002/2211-5463.12775)

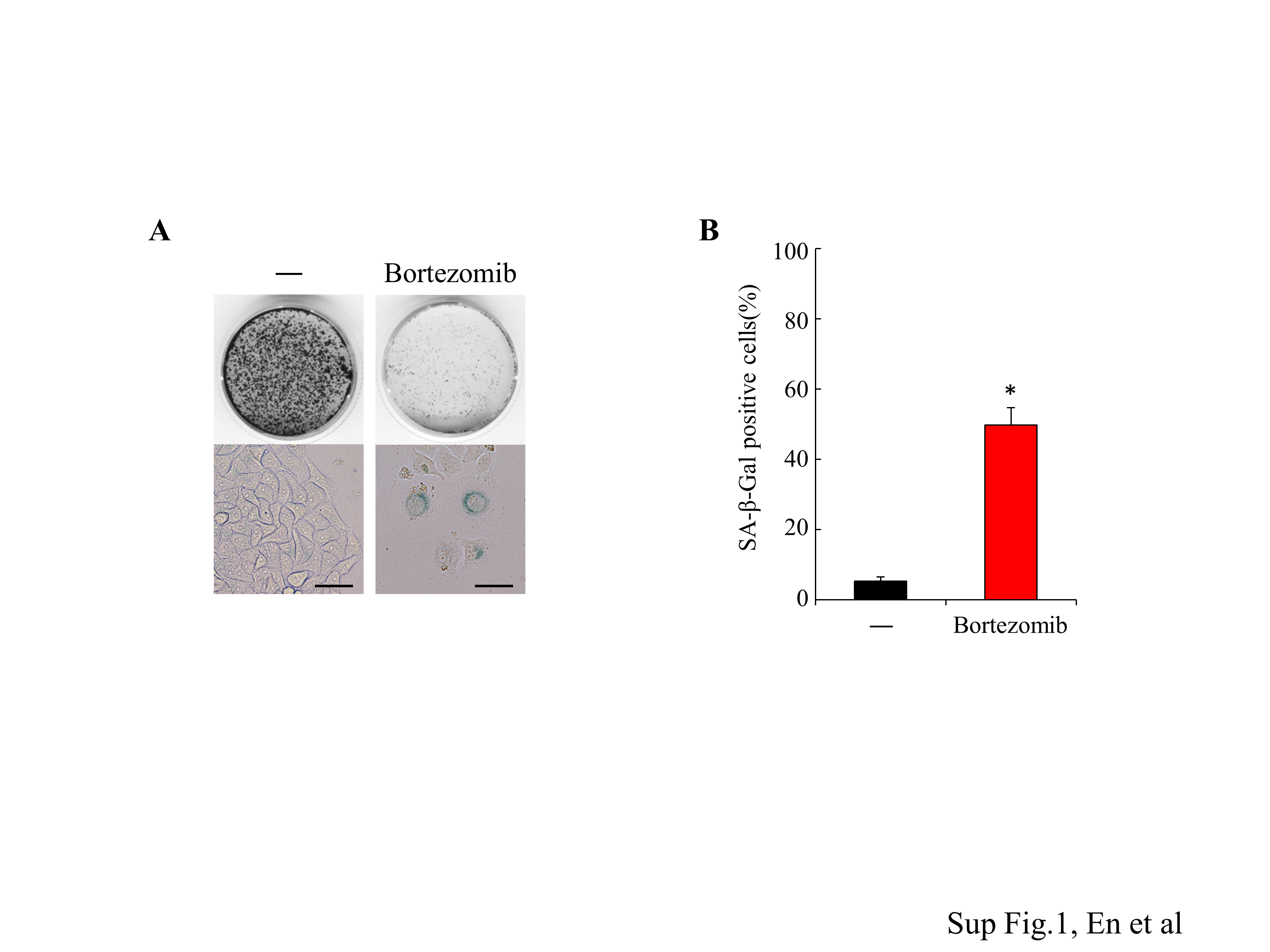

Supplement: Supplementary file 1 — Fig. S1 . Induction of cellular senescence by bortezomib in HeLa cells. (A) HeLa cells were treated with bortezomib (3 nm) for 7 days. Cell morphology was photographed (lower), and formed colonies were stained with CBB (upper). Scale bars: 50 μm. (B) The percentage of the SA‐ß‐gal‐positive cells (A) was determined (>150 cells, n = 3). An asterisk indicates statistical significance, *P < 0.05 (Student’s t‐test). Error bars indicate SD. [file FEB4-10-237-s001.tif]

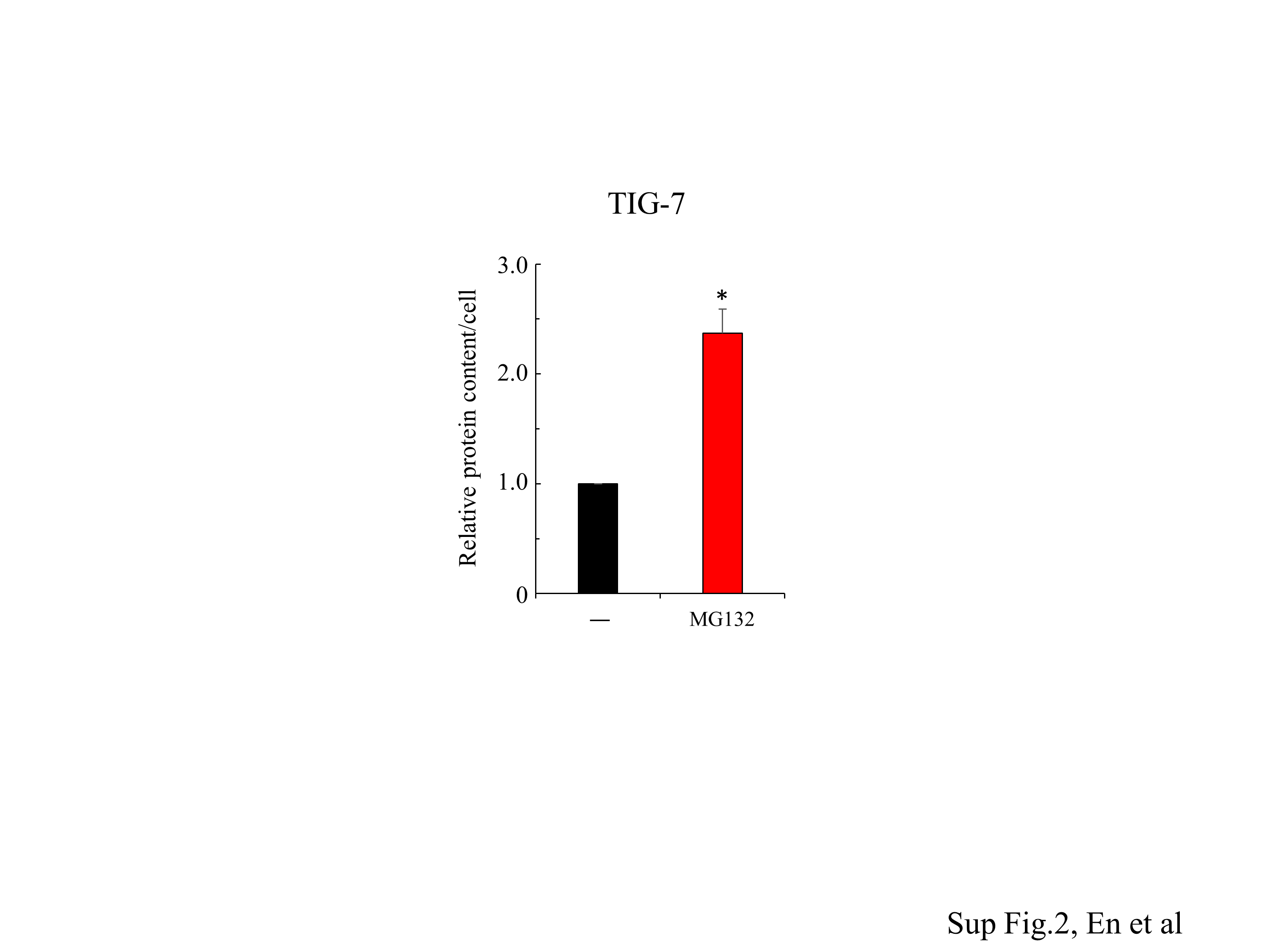

Supplement: Supplementary file 2 — Fig. S2 . Protein content in TIG‐7 cells treated with MG132. Protein content per cell was determined in TIG‐7 cells treated with MG132 (135 nm) for 4 days. Protein content is expressed as a value relative to that of the cells not treated with MG132 (n = 3). An asterisk indicates statistical significance, *P < 0.05 (Student’s t‐test). Error bars indicate SD. [file FEB4-10-237-s002.tif]

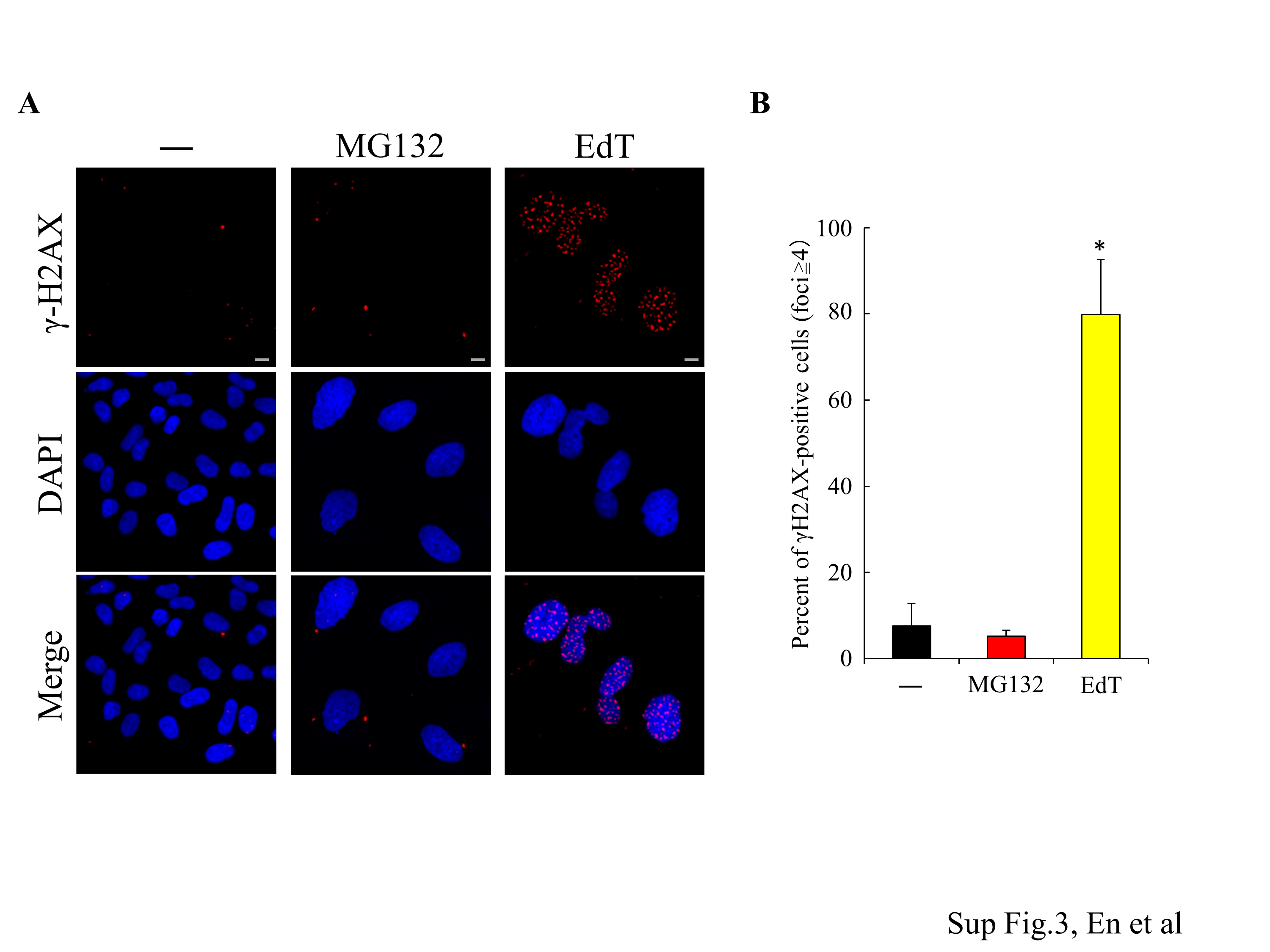

Supplement: Supplementary file 3 — Fig. S3 . DNA damage in the cells treated with MG132. (A) DNA damage was examined by immunostaining HeLa cells treated with MG132 (100 nm) or excess thymidine (EdT, 1.5 mm) for 4 days with an antibody against γ‐H2AX. DNA was stained with DAPI. Excess thymidine was used to induce DNA damage in HeLa cells. Scale bars: 10 μm. (B) The percentage of the cells with γ‐H2AX foci (A) was determined (>50 cells, n = 3). An asterisk indicates statistical significance, *P < 0.05 (one‐way ANOVA and Tukey–Kramer test). Error bars indicate SD. [file FEB4-10-237-s003.TIF]

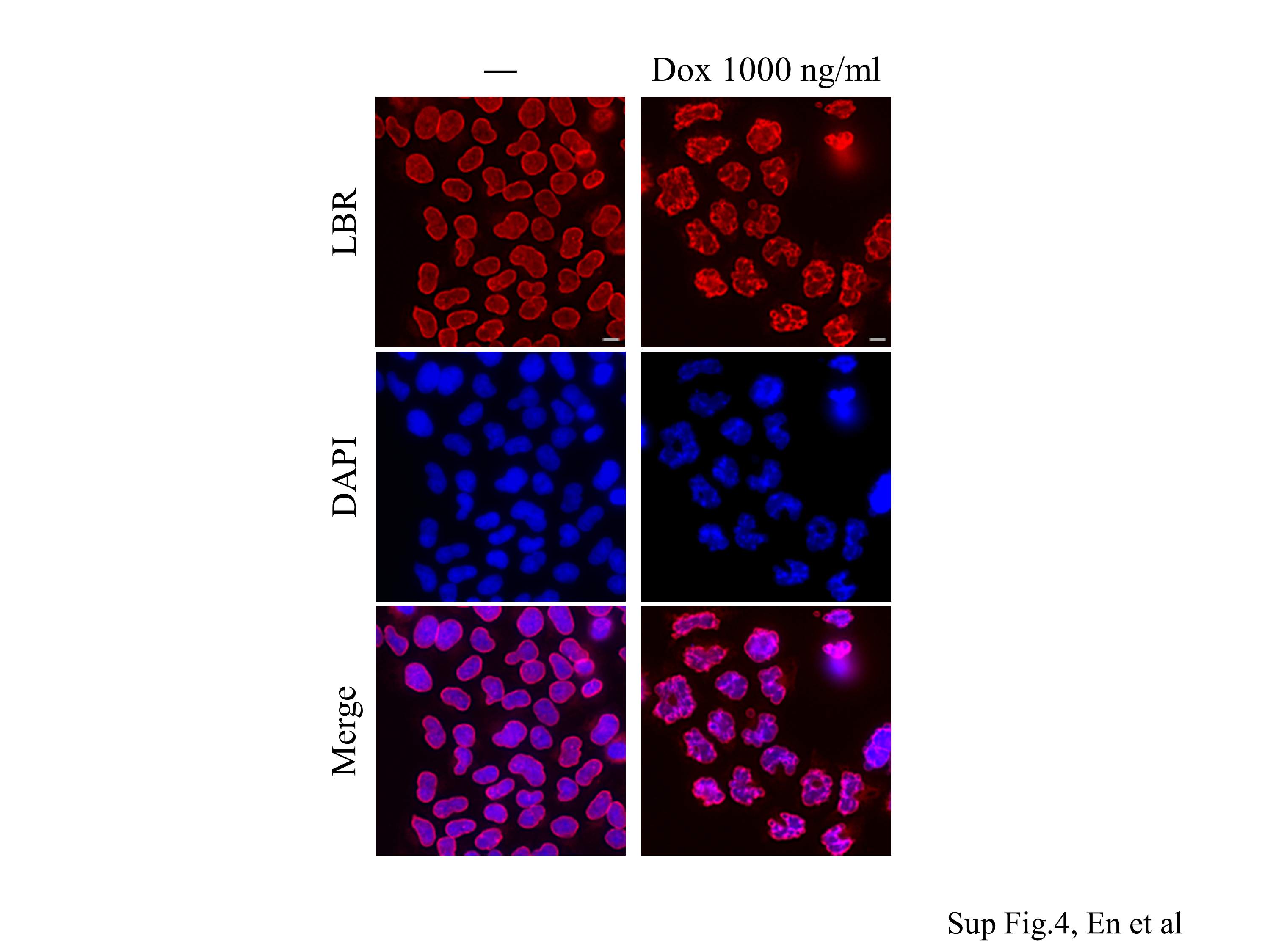

Supplement: Supplementary file 4 — Fig. S4 . Abnormal nuclear morphology induced by a high dose of Dox. Nuclear membrane was stained with an antibody against LBR in HeLaT‐LBR cells treated with Dox (1000 ng·mL−1) for 7 days. DNA was stained with DAPI. Scale bars: 10 μm. [file FEB4-10-237-s004.TIF]

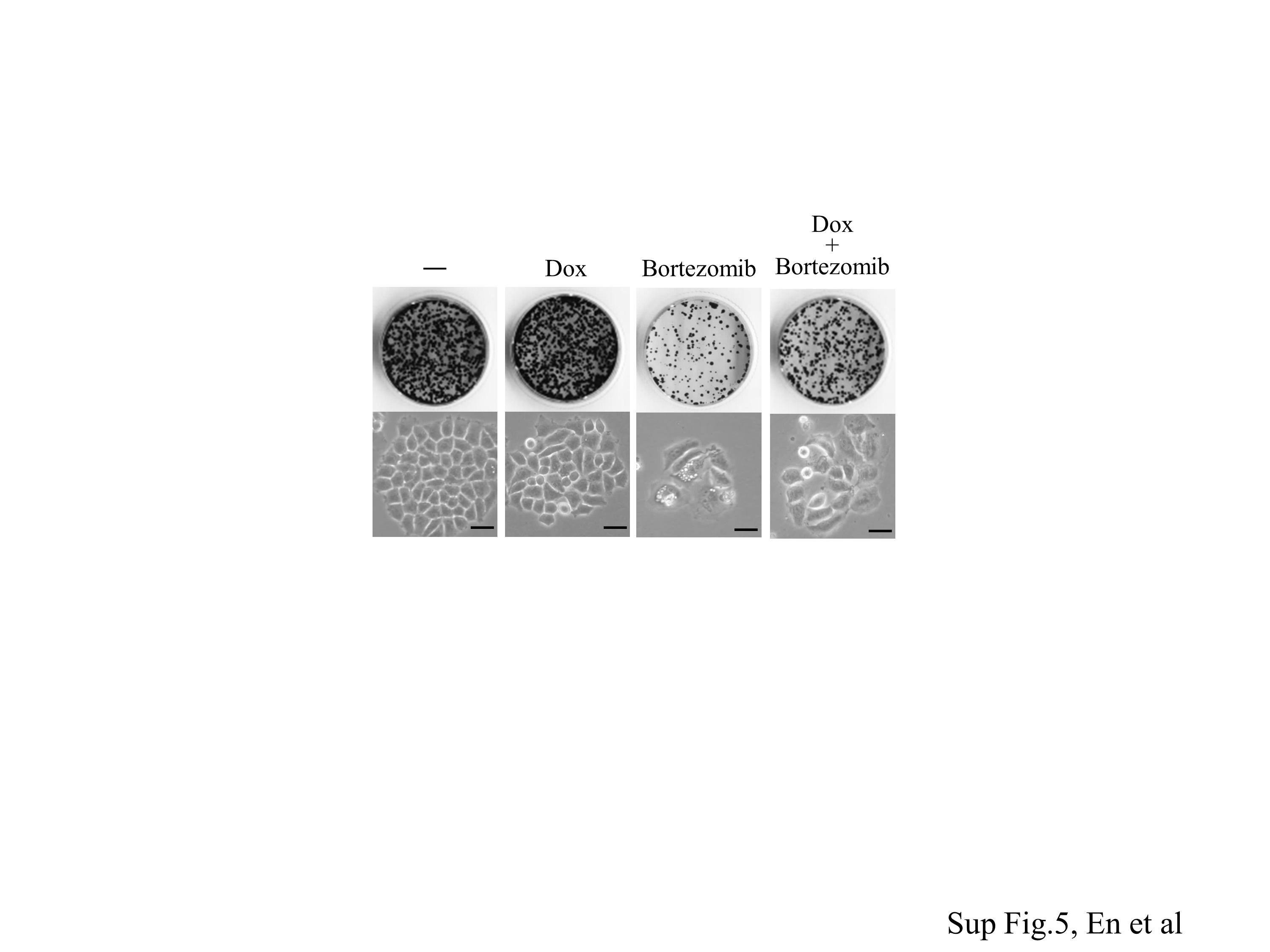

Supplement: Supplementary file 5 — Fig. S5 . Effect of enforced expression of LBR on the induction of cellular senescence by bortezomib in HeLa cells. HeLaT‐LBR cells were treated with bortezomib (3 nm) in the presence or absence of Dox (33 ng·mL−1) for 4 days, and cell morphology was photographed (lower). Cells were cultured for 9 days after replating to new dishes and then stained with CBB (upper). Scale bars: 50 μm. [file FEB4-10-237-s005.TIF]
